# Development of Abstract Word Knowledge

**DOI:** 10.3389/fpsyg.2021.686478

**Published:** 2021-06-07

**Authors:** Lorraine D. Reggin, Emiko J. Muraki, Penny M. Pexman

**Affiliations:** Department of Psychology, University of Calgary, Calgary, AB, Canada

**Keywords:** age-of-acquisition, concreteness, valence, interoception, abstract vocabulary, mouth action, affective embodiment, contextual diversity

## Abstract

The development of children’s word knowledge is an important testing ground for the embodied account of word meaning, which proposes that word meanings are grounded in sensorimotor systems. Acquisition of abstract words, in particular, is a noted challenge for strong accounts of embodiment. We examined acquisition of abstract word meanings, using data on development of vocabulary knowledge from early school to University ages. We tested two specific proposals for how abstract words are learned: the affective embodiment account, that emotional experience is key to learning abstract word meanings, and the learning through language proposal, that abstract words are acquired through language experience. We found support for the affective embodiment account: word valence, interoception, and mouth action all facilitated abstract word acquisition more than concrete word acquisition. We tested the learning through language proposal by investigating whether words that appear in more diverse linguistic contexts are earlier acquired. Results showed that contextual diversity facilitated vocabulary acquisition, but did so for both abstract and concrete words. Our results provide evidence that emotion and sensorimotor systems are important to children’s acquisition of abstract words, but there is still considerable variance to be accounted for by other factors. We offer suggestions for future research to examine the acquisition of abstract vocabulary.

## Introduction

The embodied account of word meaning proposes that children’s concepts emerge out of sensorimotor interactions (Glenberg and Gallese, 2012; Glenberg, 2015) and there is considerable evidence, particularly for concrete concepts, that this is the case (Smith et al., 2007). However, a challenge for the embodied account of word meaning, and particularly for strong accounts of embodiment, is to explain the acquisition of words that refer to abstract concepts (Borghi et al., 2017; Pexman, 2017). Specifically, if abstract word meanings are not experienced through the senses, how can children acquire them? In spite of the absence of a physical referent, children do learn the meanings of abstract words like *love* and *help*. According to a multimodal approach to word meaning, words can be learned in multiple ways, including sensory, motor, emotion, social, and linguistic information associated with the referent (Kousta et al., 2011; Borghi et al., 2019); thus providing several mechanisms by which abstract words might be acquired. The purpose of the present paper was to test these theoretical claims with a developmental approach, by examining the influence of different types of information on acquisition of abstract and concrete word meanings across childhood and into young adulthood.

Abstract words do not tend to have a clear, perceptible referent (Borghi et al., 2017; Ponari et al., 2020). They are more detached from sensorimotor experience. In addition, their meanings are less stable over time and are more influenced by life experience and situations (Barsalou et al., 2018). Brysbaert et al. (2014) described concrete words as those that can be experienced through one of the five senses (e.g., *sweet*, *jump*, *couch*) and abstract words as those for which the meaning depends on language as they cannot be experienced through the senses (e.g., *justice, dare*). Brysbaert and colleagues collected concreteness ratings for over 40,000 words, on a rating continuum ranging from very abstract (1) to very concrete (5). Many studies have used these ratings to categorize abstract words as those with concreteness ratings < 3 and concrete words as those with concreteness ratings ≥ 3 (Ponari et al., 2016, 2020; Lund et al., 2019).

### Development of Abstract Language

Ponari et al. (2016) reviewed the very limited extant research on abstract vocabulary acquisition and observed that for children between the ages of 7–8 and 9–10 years there was a surge in the quantity of abstract word meanings they understood. Ponari et al. (2016) made these observations based on subjective ratings of age-of-acquisition (age when given words are learned; AoA; Kuperman et al., 2012) and concreteness (Brysbaert et al., 2014), which were available for a set of 13,226 words. They confirmed that abstract words are, on average, rated as being acquired later than concrete words, with only 10% of a 4-year-old’s estimated vocabulary consisting of words referring to abstract concepts. They further reported that the proportion of abstract words acquired expands rapidly across middle childhood, to an estimated 40% of total vocabulary by age 12. The question of how children acquire these abstract word meanings is, as yet, unanswered, but there are two main proposals that have so far been tested in developmental studies. We review these next.

### Acquisition of Abstract Vocabulary – Specific Theoretical Proposals

#### Affective Embodiment Account

The *affective embodiment account* (Kousta et al., 2011; Borghi et al., 2017) provides a way in which the meanings of abstract words could be grounded in bodily experience and thus is consistent with the broad notion of embodiment. The proposal is that emotional experience is key to the grounding of the representation of abstract concepts (Kousta et al., 2011). By the affective embodiment account, the emotion aspects of word meanings provide a way for children to begin to build representations of abstract concepts. The meanings of children’s first abstract words may be grounded in felt experience, such as associating *love* with a feeling of being cuddled. Emotion words can provide children with “essence placeholders” (Shablack et al., 2020, p. 1538) that categorize their bodily experiences.

There is evidence to support the claim that abstract words are grounded in emotional experience. Altarriba et al. (1999) noted that the valence of words (positive, neutral, negative) interacted with concreteness and found that although valenced words were rated low on concreteness (like other abstract words), they were rated high on imageability and context availability. Relatedly, the results of a semantic categorization study with adults demonstrated that emotion information facilitated processing of abstract words, whereas sensorimotor information facilitated processing of concrete words (Newcombe et al., 2012). Similarly, Pexman and Yap (2018) found that valence information (positive or negative) facilitated adults’ semantic decisions to abstract words, but not to concrete words.

##### Valence

There are many aspects to emotional experience, but the developmental research has thus far tended to focus on valence information. Evidence that valence may be related to vocabulary acquisition, and may be particularly important for abstract vocabulary acquisition, was reported in previous studies that have analyzed AoA norms (Kousta et al., 2011; Ponari et al., 2016). Kousta et al. (2011) reported that abstract valenced words (regardless of polarity) were rated as being acquired earlier than abstract words that were not valenced (neutral words). Using different AoA norms, Ponari et al. (2016) confirmed the relationship between concreteness (Brysbaert et al., 2014) and AoA (Kuperman et al., 2012) across more than 13,000 words. That is, Ponari et al. (2016) confirmed that abstract words are rated to be acquired later than concrete words. Importantly, they also found an interaction between valence (Warriner et al., 2013) and concreteness for AoA ratings. They found that for abstract words in particular, those that are valenced (both positive and negative) were rated as being acquired earlier than those that are neutral.

Ponari et al. (2016) also examined the processing of abstract and concrete words in an auditory lexical decision task with children aged 6 to 12 years. They found that valence affected response accuracy specifically for abstract words, and only in children aged 8–9 years. They did not find valence effects in the younger children (6–7 years) and noted that response accuracy for the younger children was very low, suggesting the children did not know many of the words. Further, valence effects were not found in the responses of the older children (10–11 years); this was attributed to increased knowledge of neutral abstract word meanings in this older group. The authors concluded that their results were consistent with the proposal that emotion (as captured by the valence dimension) provides a bootstrapping mechanism for learning the meanings of abstract words. Similarly, in a recent vocabulary learning experiment, Ponari et al. (2020) found that 7–9 year old children were able to provide more accurate definitions for valenced words than neutral words.

In a closely related study, Lund et al. (2019) further tested the predictions of the affective embodiment account by investigating valence and concreteness effects in children’s reaction times on an auditory lexical decision task. Their participants were children aged 5-, 6- and 7-years. They found a facilitatory effect of valence in the responses of both 6- and 7-year-olds. This sensitivity to emotional information was not present in the 5-year-olds. There was also an interaction of valence and concreteness in the responses of the 6-year-olds, which involved a processing advantage for positive abstract words compared to neutral abstract words, providing some limited support for the affective embodiment prediction that emotion information plays a stronger role in the processing of abstract words than concrete words. Lund et al. (2019) found effects of valence at an earlier age than in the study by Ponari et al. (2016), suggesting that valence information might support children’s early acquisition of abstract vocabulary.

Finally, Kim et al. (2020) tested a different cognitive process, children’s recognition memory, for the interaction of valence and concreteness. They presented 7- to 8-year-old children with spoken word stimuli that varied on both valence and concreteness. In a recognition memory test the same day, they assessed children’s accuracy in identifying the words they had heard earlier. They found the predicted interaction of valence and concreteness in children’s recognition memory accuracy: children were more accurate in their memory for negative words than for neutral words, but only for abstract meanings; valence had no effect on memory for concrete meanings. Thus, the Kim et al. findings were consistent with those described above; all of these findings have been taken as support for the predictions of affective embodiment, wherein children are proposed to ground abstract word meanings in emotional systems.

The findings from the behavioral studies reviewed above do provide some support for the affective embodiment account, but there are certainly limitations to this work, and other aspects of the findings suggest that the picture may be more complex. First, in order for Lund et al. (2019) to provide words that were known to young children, the ‘abstract’ words selected for the study were relatively less abstract than those presented in other studies (e.g., Ponari et al., 2016). Secondly, although Ponari et al. (2020) found evidence of valence in the definition task they used with 7–9 year old children, they also found no differences in accuracy for valenced versus neutral words on an auditory lexical decision task, and there was no impact of valence in the definitions provided by the 9–10 year-old children. Vigliocco et al. (2018) noted that valence information does not appear to support abstract vocabulary acquisition beyond the age of 9. The authors concluded that this is evidence of a hybrid or multimodal view of semantic representation, with both embodied and linguistic features. Third, the developmental studies conducted so far have examined the construct of emotion only in terms of valence information. Yet there is much more to emotional experience than the evaluative aspect captured by valence. In the present study, we investigated the roles of several other aspects of emotional experience in development of abstract word knowledge.

##### Arousal

Arousal is proposed to be a dimension of emotional experience, involving the degree of excitement or intensity associated with word meaning (Russell, 1980). The effects of arousal on children’s abstract vocabulary acquisition have not yet been examined. but the dimension might capture some of the more visceral aspects of emotional experience than those associated with valence.

##### Interoception

Similarly, interoception captures the various sensations *inside* the body (Barsalou, 1999; Barsalou and Wiemer-Hastings, 2005; Borghi et al., 2017; Connell et al., 2018; Harpaintner et al., 2018). To test the proposal that abstract concepts are grounded through interoception, Connell et al. (2018) examined a large set of modality-specific sensorimotor experience ratings (an early iteration of the Lancaster Sensorimotor Norms, Lynott et al., 2020) and compared strength of modality-specific experience for abstract and concrete words. They found that interoceptive strength ratings were higher for abstract than concrete word meanings and concluded that interoception is more important to the representations of abstract concepts than concrete concepts. Some, but not all, of this difference in interoceptive strength between abstract and concrete words could be attributed to the fact that emotions, which tend to be abstract, have higher interoception ratings. Similarly, Zdrazilova et al. (2018) used a face-to-face task to explore the words and gestures that people used to communicate abstract and concrete word meanings. They found that interoceptive states were frequently referenced as participants described abstract (but not concrete) meanings.

##### Mouth Action

In addition, Anna Borghi and colleagues have proposed that the mouth motor system, by virtue of its fundamental role in both overt and covert language production, is important to abstract meanings (e.g., Granito et al., 2015; Barca et al., 2017; Borghi, 2020). For instance, Barca et al. (2020) conducted a semantic categorization task with children in grade 3 (approximately 8–9 years of age) with concrete, abstract, and emotion word stimuli. The results showed that children who had used a pacifier extensively in early childhood were particularly slow to respond to abstract words. The inference was that extensive pacifier use disrupts mouth-associated social and linguistic experiences, and that these are important to abstract vocabulary acquisition. Thus, Barca et al. concluded that mouth effectors are important for abstract vocabulary acquisition (see also Barca et al., 2017).

##### Head Action

It is also possible that head actions (distinct from actions of the mouth/throat) tend to be engaged in emotions and when experiencing the meanings of abstract words, because many abstract meanings involve social, cognitive, and internal states and relations that are likely to be affected through movements of the head and eyes. Indeed, Connell (2021; see also Banks and Connell, 2021); reported that head (non-mouth) action strength was important to the representations derived for many abstract concepts from the Lancaster Sensorimotor Norms. There is also some neuroimaging evidence to suggest that abstract word meaning may be grounded to some extent in head-related sensorimotor experience. Dreyer and Pulvermüller (2018) identified stronger activation in motor regions associated with the face during passive reading of abstract mental words (e.g., *logic*) relative to activation in motor regions associated with hand actions. As such, head action strength might also be important for abstract vocabulary acquisition; however, the association between head action and abstract word representation has yet to be tested developmentally.

Thus, there are several ways in which emotional experience could be measured, beyond valence, and by which the affective embodiment account could be tested more fully. In addition, Borghi et al. (2017) noted that affective embodiment accounts for the grounding of valenced abstract concepts but does not provide a clear explanation for representation of abstract concepts that do not have emotional connotations. Therefore, there is a need for research that considers other mechanisms, beyond emotion, that might support word learning into adolescence and adulthood. One other mechanism that has been proposed to support abstract vocabulary acquisition is language experience.

### Learning Through Language Proposal

There are numerous proposals that language is particularly important to the meanings of abstract concepts (e.g., Paivio, 1991; Borghi et al., 2019; Borghi, 2020; Dove et al., 2020). In the developmental context, it has been proposed that abstract words are learned through linguistic cues. For instance, Gleitman and colleagues (Gleitman, 1990; Gleitman et al., 2005; Papafragou et al., 2007) proposed the *syntactic bootstrapping hypothesis*, by which syntactic information is used to support word learning. Gleitman (1990) proposed that children initially learn words through a word-to-world mapping, pairing a word with a referent in the environment. Once children have acquired knowledge about regularities in language they begin to infer word meanings from linguistic context, a ‘structure-to-world’ pairing which is particularly helpful for learning abstract words, since these tend not to have observable referents. The proposal of the syntactic bootstrapping hypothesis is that children can only learn abstract words after they have enough sophisticated language knowledge to match an event with the appropriate syntactic structure.

In addition to syntactic cues, children may also learn word meanings from situational context (Shablack et al., 2020) and from regularities in the ways words are used and co-occur in language (Andrews et al., 2009; Hills et al., 2010). The proposal that abstract words are learned through language requires that children must have first learned at least some concrete concepts with which to ground the meaning of new abstract words that do not have a sensorimotor component. This co-occurrence or distributional approach does not emphasize the need to develop specific syntactic structures with which to scaffold later learning, but rather proposes that linguistic knowledge can provide an additional grounding for word meaning in the absence of experiential sensory, motor, or emotion information.

Lund et al. (2019) provided some evidence for the role of language in abstract vocabulary acquisition. They found that in an auditory lexical decision task, children’s response times to neutral (non-valenced) abstract words were related to the children’s language skills, such that children with more advanced language skills responded more quickly to neutral abstract words than did children with weaker language skills. They took this as evidence for what they termed the *language competence hypothesis*: the proposal that when words do not have the benefit of additional information from sensory, motor, or emotion attributes children draw upon language experience to learn those word meanings. Vigliocco et al. (2009) also predicted that language experience, and in particular distributional information, may be more important for abstract words than for concrete words, since abstract meanings lack sensory and motor contingencies. Similarly, Ponari et al. (2020) suggested that the results of their word learning experiment provided evidence of learning through language. They found that while 9- to 10-year-old children learned the meanings of new abstract words they did not show evidence of experiential benefit (i.e., through emotion) in their meaning definitions. In contrast, valence played a role in the definitions provided by younger children (aged 7- to 9 years), who could define valenced abstract words more accurately than neutral abstract words.

Another aspect of language experience that may be important to children’s abstract vocabulary acquisition is the diversity of contexts in which words are experienced. Hills et al. (2010) investigated why children learn some words earlier than others. They examined the diversity of contexts in the learning environment and found that a word’s contextual diversity – the number of unique word types with which a word co-occurs in the child’s language environment – predicted the order of acquisition. They found that early word acquisition was influenced by *preferential acquisition*, in which a word is more likely to be learned if it is in close proximity to many other words in the learning environment. Secondly, they found that word learning was influenced by the *lure of associates*: a word was more likely to be learned if it is related to other words the child already knows. The lure of associates principle is consistent with the proposal that abstract words are acquired through language. If first (concrete) words are learned through observation and grounded through links to sensory, motor, and emotion experiences, later words can then be grounded through language, via the lure of associates. Hills et al. did not examine the effects of contextual diversity for acquisition of abstract words specifically, but we did so in the present study.

### This Paper

The purpose of the present paper was to test these various proposals about factors that are important to children’s abstract vocabulary acquisition, using a large-scale vocabulary acquisition dataset that spans early school to University ages (Dale and O’Rourke, 1981; as updated in Brysbaert and Biemiller, 2017). We tested whether each of the following factors predict vocabulary acquisition: valence, arousal, interoceptive strength, mouth action strength, head action strength, and diversity of language context. We also tested whether each of these factors interacts with concreteness, since each proposal holds that the factor should have stronger effects for abstract than concrete word acquisition.

## Materials and Methods

The methodology of the present study involved analyses using five existing datasets. The dependent measure in our analyses was the test-based age of acquisition (AoA) data that were originally reported by Dale and O’Rourke (1981). The Dale and O’Rourke (1981) data estimated AoA by objective means, testing children’s vocabulary knowledge across school grades and including over 31,000 unique words. Each word’s AoA in those data is equal to the lowest grade in which it is known to an estimated 50–70% of students, based on children’s responses to three-alternative multiple-choice tests and corrected for guessing. Brysbaert and Biemiller (2017) updated and expanded the Dale and O’Rourke (1981) data so that they offered vocabulary estimates for grades 2, 4, 6, 8, 10, 12, 13, and 16 (the latter two are university levels). Items in the Brysbaert and Biemiller (2017) dataset may occur more than once with different ages, reflecting when different meanings of a word were acquired. In these cases, we used the AoA of the earliest acquired meaning in our analysis.

Our predictor variables included two control variables, word frequency, which was included in analyses to control for the known relationship between frequency and AoA (log subtitle frequency; Brysbaert and New, 2009) and word length. In addition, we had seven key semantic predictors: ratings of concreteness (the degree to which a word refers to something that can be experienced through one of the five senses; Brysbaert et al., 2014), ratings of word valence (the degree to which reading a word makes you feel unhappy or happy) and arousal (the degree to which reading a word makes you feel calm or excited; Warriner et al., 2013), three measures from the Lancaster Sensorimotor Norms: ratings of interoceptive strength (the degree to which a concept is experienced by internal sensations of the body), mouth action strength (the degree to which a concept is experienced by mouth/throat actions) and head action strength (the degree to which a concept is experienced by head actions excluding the mouth; Lynott et al., 2020), and semantic diversity (the extent to which a word appears in diverse contexts; Hoffman et al., 2013).

## Results

We extracted test-based age of acquisition norms from Brysbaert and Biemiller (2017), which were derived from the data in Dale and O’Rourke (1981) Living Word Vocabulary, concreteness ratings (Brysbaert et al., 2014), valence and arousal ratings (Warriner et al., 2013), semantic diversity ratings (Hoffman et al., 2013), interoceptive, head, and mouth strength ratings (Lynott et al., 2020), log subtitle frequency (Brysbaert and New, 2009) and length. In total there were 9,916 items for which we had values for all variables of interest. We calculated correlations between all variables of interest, as well as the variables of positive valence (i.e., all variables with valence greater than or equal to 5), negative valence (i.e., all variables with valence less than or equal to 5) and valence extremity (i.e., the absolute value of the valence rating from 5, the neutral point on the scale). All variables were significantly correlated (*p* < 0.01) with the exception of head and mouth perceptual strength, which were not correlated with test-based AoA, nor was mouth action strength correlated with valence (Table 1).

**TABLE 1:** Means, standard deviations, and correlations of all variables of interest.

**Variable**	***M***	***SD***	**1**	**2**	**3**	**4**	**5**	**6**	**7**	**8**	**9**	**10**	**11**	**12**
(1) Test-based AoA	8.63	4.04												
(2) Length	7.48	2.35	0.23**											
(3) Frequency	1.73	0.92	−0.60**	−0.36**										
(4) Concreteness	3.21	1.05	−0.32**	−0.29**	0.10**									
(5) Valence	5.09	1.27	−0.18**	–0.02	0.20**	0.09**								
(6) Positive Valence	5.96	0.70	−0.19**	0.04**	0.23**	−0.13**	1.00**							
(7) Negative Valence	3.92	0.83	0.03*	−0.08**	−0.10**	0.18**	1.00**	NA						
(8) Valence Extremity	1.03	0.75	−0.10**	0.06**	0.15**	−0.16**	−0.08**	1.00**	1.00**					
(9) Arousal	4.19	0.89	0.03**	0.10**	0.04**	−0.17**	−0.17**	0.28**	−0.42**	0.35**				
(10) Interoceptive Strength	1.03	0.90	0.03**	0.07**	0.10**	−0.41**	−0.11**	0.36**	−0.40**	0.38**	0.31**			
(11) Mouth Action Strength	1.29	0.94	–0.01	0.03**	0.10**	−0.18**	0.03**	0.22**	−0.20**	0.21**	0.14**	0.28**		
(12) Head Action Strength	2.29	0.73	−0.06**	0.11**	0.14**	−0.19**	0.09**	0.26**	−0.21**	0.22**	0.15**	0.25**	0.25**	
(13) Semantic Diversity	1.56	0.35	−0.21**	−0.04**	0.39**	−0.38**	0.04**	0.07**	–0.02	0.05**	0.02	0.19**	0.07**	0.09**

M and SD are used to represent mean and standard deviation, respectively. AoA, Age of Acquisition. *Indicates *p* < 0.05. **Indicates *p* < 0.01.

We tested theories of abstract word acquisition with a hierarchical regression model. In the first stage we entered all predictors of test-based AoA. Predictors in the first stage accounted for 42.63% of variance in test-based AoA. We observed significant effects for all predictors with the exception of length and head and mouth action strength (Table 2). We then added interactions between concreteness and each semantic predictor variable to assess the affective embodiment account and learning through language theories of abstract word acquisition. There was a significant improvement in model fit with the addition of the interactions (Table 2), with the interactions accounting for an additional 0.75% of variance in test-based AoA.

**TABLE 2:** Hierarchical regression predicting test-based AoA using all variables of interest (*N* = 9,916).

**Variables**	***b***	***b* 95% CI [LL, UL]**	***sr*^2^**	**Fit**	**Difference**
Intercept	6.24**	[6.19, 6.29]			
Length	0.02	[−0.04, 0.08]	0.000		
Frequency	−1.47**	[−1.54, −1.40]	0.109		
Concreteness	−1.45**	[−1.52, −1.38]	0.090		
Valence - Linear	1.79**	[1.42, 2.17]	0.005		
Valence - Quadratic	−2.01**	[−2.39, −1.63]	0.006		
Arousal	0.11**	[0.06, 0.17]	0.001		
Interoceptive Strength	−0.13**	[−0.19, −0.06]	0.001		
Mouth Action Strength	–0.05	[−0.10, 0.01]	0.000		
Head Action Strength	–0.01	[−0.07, 0.05]	0.000		
Semantic Diversity	−0.48**	[−0.54, −0.41]	0.012		
				*R^2^* = 0.426**	
Intercept	6.36**	[6.29, 6.42]			
Length	0.02	[−0.04, 0.08]	0.000		
Frequency	−1.47**	[−1.54, −1.41]	0.109		
Concreteness	−1.42**	[−1.49, −1.35]	0.085		
Valence - Linear	1.48**	[1.10, 1.86]	0.003		
Valence - Quadratic	−1.71**	[−2.09, −1.33]	0.004		
Arousal	0.09**	[0.03, 0.15]	0.001		
Interoceptive Strength	−0.07*	[−0.14, −0.00]	0.000		
Mouth Action Strength	−0.10**	[−0.16, −0.04]	0.001		
Head Action Strength	0.02	[−0.05, 0.08]	0.000		
Semantic Diversity	−0.48**	[−0.55, −0.42]	0.012		
Concreteness by Valence - Linear	−1.56**	[−1.95, −1.16]	0.003		
Concreteness by Valence - Quadratic	1.48**	[1.10, 1.87]	0.003		
Concreteness by Arousal	–0.00	[−0.06, 0.06]	0.000		
Concreteness by Interoceptive Strength	0.13**	[0.05, 0.20]	0.001		
Concreteness by Mouth Action Strength	0.06*	[0.01, 0.12]	0.000		
Concreteness by Head Action Strength	0.01	[−0.05, 0.07]	0.000		
Concreteness by Semantic Diversity	0.00	[−0.06, 0.06]	0.000		
				*R^2^* = 0.434**	Δ*R^2^* = 0.007**

A significant *b*-weight indicates the semi-partial correlation is also significant. *b* represents unstandardized regression weights. *sr*^2^ represents the semi-partial correlation squared. *LL* and *UL* indicate the lower and upper limits of a confidence interval, respectively. *Indicates *p* < 0.05. **Indicates *p* < 0.01.

**FIGURE 1:**
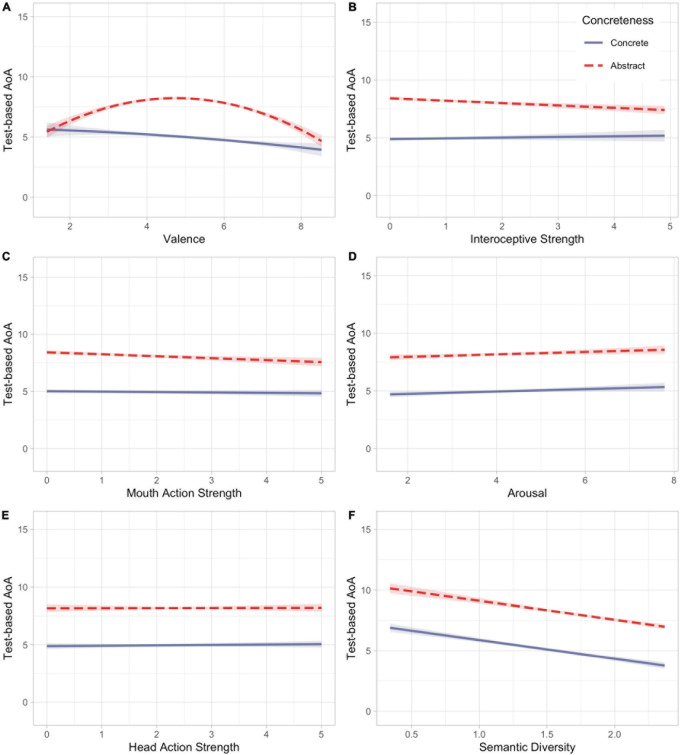
AoA, Age of Acquisition. Plots depict partial residuals of the interaction between concreteness and **(A)** Valence, **(B)** Interoceptive Strength, **(C)** Mouth Action Strength, **(D)** Arousal, **(E)** Head Action Strength and **(F)** Semantic Diversity in predicting AoA. All plots represent the interaction relationship when other variables in the model are held constant.

First, the affective embodiment account was tested via an interaction between concreteness and valence in predicting test-based AoA. Valence was entered as a linear and a quadratic term due to the bipolar nature of the scale (e.g., 1 = negative, 9 = positive, 5 = neutral). We observed a significant interaction between concreteness and the quadratic term of valence on test-based AoA while holding all other parameters constant, *b* = 1.48, *t*(9898) = 7.54, *p* < 0.001, such that neutral abstract words were learned significantly later than neutral concrete words. The interaction between concreteness and valence on test-based AoA is depicted in Figure 1A.

To further test aspects of emotional experience that could be related to abstract word acquisition, we examined interactions between concreteness and arousal, interoceptive strength, head action strength, and mouth action strength. We observed a significant interaction between concreteness and interoceptive strength on test-based AoA while holding all other parameters constant, *b* = 0.13, *t*(9898) = 3.46, *p* = 0.001, such that abstract words with low interoceptive strength were learned later than concrete words with low interoceptive strength; abstract words with high interoceptive strength were not acquired significantly later than concrete words with high interoceptive strength. The interaction between concreteness and interoceptive strength on test-based AoA is depicted in Figure 1B.

There was also a significant interaction between concreteness and mouth action strength on test-based AoA while holding all other parameters constant, *b* = 0.06, *t*(9898) = 2.15, *p* = 0.031, such that abstract words with lower mouth action strength were learned later than concrete words with lower mouth action strength; abstract words with higher mouth action strength were not acquired significantly later than concrete words with higher mouth action strength. The interaction between concreteness and mouth action strength on test-based AoA is depicted in Figure 1C.

We observed no significant interaction between concreteness and arousal on test-based AoA while holding all other parameters constant, *b* = −0.00, *t*(9898) = −0.05, *p* = 0.959 (see Figure 1D). Nor was there a significant interaction between concreteness and head action strength, *b* = 0.01, *t*(9898) = 0.32, *p* = 0.746 (see Figure 1E).

Finally, we tested the learning through language proposal via an interaction between concreteness and semantic diversity in predicting test-based AoA. We observed no significant interaction between concreteness and semantic diversity, *b* = 0.00, *t*(9898) = 0.08, *p* = 0.936 (see Figure 1F).

## Discussion

The purpose of the present study was to investigate the degree to which acquisition of abstract word knowledge across childhood and into young adulthood is related to the emotional, sensorimotor, and linguistic information associated with a word’s referent. We tested specific proposals for how abstract words are acquired. According to the affective embodiment hypothesis, emotional experience is key to learning the meanings of abstract words (Kousta et al., 2011) because many abstract words refer to internal states that can create an emotional experience (Ponari et al., 2016). According to the learning through language proposal, abstract word meanings are acquired through experience with language.

In the present study we examined several aspects of emotional experience that could be relevant to the acquisition of abstract words. Kousta et al. (2011) found that valence alone does not capture completely the meaning of abstract words. Therefore, in addition to valence, we expanded the emotional properties to include measures of word arousal and interoceptive strength. To examine associated sensorimotor experiences we examined mouth and head action strength. Therefore, the affective embodiment account was tested by examining the relationship between word valence and word arousal with test-based AoA, and also by testing whether these relationships varied for abstract and concrete words. We observed a significant quadratic relationship between valence and test-based AoA for abstract words, suggesting that emotional information (both positive and negative) is more important for abstract word acquisition than for concrete word acquisition. Furthermore, we observed a significant interaction between interoception strength and concreteness on test-based AoA, such that abstract words associated with less interoceptive experience are acquired later than concrete words with less interoceptive experience, whereas there is less difference in the acquisition of abstract and concrete words associated with more interoceptive experience. Additionally, we observed a significant interaction between mouth action strength and concreteness on test-based AoA, with abstract words that had lower mouth action strength ratings being acquired later than concrete words with lower mouth action strength ratings, and less difference in the acquisition of abstract and concrete words with more mouth action strength. This would suggest that mouth effectors are more important for abstract word acquisition than for concrete word acquisition, consistent with arguments made by Borghi and colleagues (Barca et al., 2017, 2020; Borghi et al., 2019) about the role of mouth experience in grounding abstract word meanings. Thus, valence, interoceptive strength, and mouth action strength facilitate acquisition of abstract words, and are less important for acquisition of concrete words, consistent with the predictions of the embodiment account. These results provide further evidence to support the claim that abstract words are grounded in emotional and associated sensorimotor experiences.

Borghi et al. (2017) argued that one limitation of the affective embodiment account is that the proposed role of valence does not account for all the mechanisms underlying the acquisition of abstract concepts. The current analysis shows that additional conceptualizations of emotional experience, namely interoceptive and mouth action strength, can further explain the development of abstract word meanings as grounded in a more broadly defined operationalization of emotion. In addition, as proposed in Borghi and Binkofski (2014) *words as social tools* (WAT) view, we found that abstract concept acquisition is associated with mouth action strength. Borghi et al. (2017) proposed that the mechanism of subvocalization is more important for abstract than concrete words, as evidenced in the current analysis by increased mouth action strength for abstract words.

The learning through language proposal would suggest that the meanings of abstract words and concepts can be acquired through language experience. This proposal is consistent with both the distributional theory of semantics (Andrews et al., 2009) and the syntactic bootstrapping hypothesis (Gleitman et al., 2005). It was predicted that linguistic distribution information may be more important for abstract words than for concrete words, since abstract meanings lack sensory and motor contingencies (Vigliocco et al., 2009). We tested the specific notion that contextual diversity (operationalized here as the semantic diversity variable computed by Hoffman et al., 2013) would facilitate abstract vocabulary acquisition more than concrete vocabulary acquisition. We observed a significant relationship between semantic diversity and AoA, but this relationship did not vary for abstract and concrete words. Therefore, consistent with the findings from Hills et al. (2010), early word acquisition was influenced by preferential acquisition, in that words, both concrete and abstract, are learned earlier when they are present in more diverse contexts in the learning environment. This suggests that exposure to word meanings in diverse linguistic contexts is important to both abstract and concrete word acquisition. As contextual diversity did not disproportionately affect the acquisition of abstract words, the current findings did not provide evidence for the view that abstract word learning was influenced by lure of associates, or that abstract words are more likely to be learned if they are related to other words the child already knows. Rather, the current findings suggest that all words, both concrete and abstract, are learned earlier when they have been experienced in diverse contexts, presumably because their meanings have greater opportunities to be linked to already known words.

In the *language and situated simulation* (LASS; Barsalou et al., 2008) multiple representations view, both simulated modal (sensory, motor, and emotion) and linguistic systems can support learning. The current findings are consistent with this view, in that emotion systems are important to the learning of abstract words, and linguistic systems support both concrete and abstract word learning. Notably, we did not test whether other aspects of language use, or acquisition of specific language structures (e.g., relational terms) might be particularly important to abstract vocabulary acquisition. This will be an important issue for future research. We turn to other such topics next.

### Limitations and Future Directions

One limitation of our study is that we examined AoA starting at grade 2. Therefore, there is data missing from our analysis concerning the very early stages of language development. There is evidence that most children have acquired at least some emotion words by age 2 (Wellman et al., 1995) and theories of acquisition will need to account for this early acquisition. To address this limitation, we examined the characteristics of children’s earliest abstract words, based on information extracted from the Wordbank database^1^. Wordbank provides information about vocabulary acquisition for children under age 3 (Frank et al., 2017). The database provides vocabulary norms and aggregate data on the proportion of children at a particular age who know a specific word, based on over 42,000 administrations of the MacArthur-Bates Communicative Development Inventories (CDIs). The CDIs are widely used parent-report instruments to gather data about early language acquisition. Of the nearly 600 words in this database, 96 had concreteness ratings < 3, based on the Brysbaert et al. (2014) concreteness norms. Thus, these are children’s earliest abstract words. Interestingly, none of these 96 words were nouns. Instead, children’s early-acquired abstract words were closed-class words including determiners (e.g., *the, all*), conjunctions (e.g., *and, or*), prepositions (e.g., *for, to, by, with*), exclamations (e.g., *yes, no, hi, bye*), and pronouns (e.g., *this, that, they, it*); as well as open-class words including adverbs (e.g., *there, away, how, now, why, better*), adjectives (e.g., *yucky, careful, pretty*), and verbs (e.g., *be, like, think*). We used ratings from Warriner et al. (2013) to examine the valence of these early abstract words. Valence ratings were available for 42 of these words, including seven words with negative valence ratings, 18 words with neutral valence ratings, and 17 words with positive valence ratings. In sum, children do present with knowledge of a variety of abstract words before age 3, including several that do not have associated emotion information, suggesting that acquisition of these earliest words is not entirely explained by affective embodiment. Those early abstract words that are associated with emotion information tend not to be negatively valenced. Indeed, there is evidence that children tend to hear more positive than negative words in child-directed speech which may reflect a tendency of parents and caregivers to avoid negative language with children, especially in the early years (Ponari et al., 2016).

As described in the Results section above, the omnibus model accounted for 43.4% of variance in test-based AoA, implying that while we have identified some of the variables that predict vocabulary acquisition, there is still considerable variance to be accounted for by other factors. One possible explanation concerns the heterogeneity of both abstract and concrete concepts; for instance, abstract words refer to a wide variety of concepts, including words that refer to emotions, mental states, interospections, social concepts, etc. (Kiefer and Harpaintner, 2020; Muraki et al., 2020b). Desai et al. (2018) proposed four categories of abstract words: numerical, emotional, morality, and theory of mind. Similarly, Borghi et al. (2019) also provided evidence for four types of abstract words: philosophical/spiritual, physical/spatial/quantitative, self-sociality, and emotive/inner states. In two recent studies, Muraki and colleagues found that different types of abstract verbs can be distinguished in terms of associated behavioral and neural responses (Muraki et al., 2020a, b). The inference is that different types of abstract words are associated with different combinations of linguistic, sensory, emotion, and other information. We did not attempt to capture this variability in the present analyses, but it is an important topic for future research.

While the abstract/concrete distinction has historically been conceptualized as a dichotomy, there is considerable evidence that it is more accurately characterized as a continuum. By the affective embodiment account, emotion concepts are assumed to contain both abstract and concrete elements and indeed ratings of emotion concepts suggest that they tend to fall more toward the middle of the abstract-concrete continuum (Altarriba and Bauer, 2004; Winkielman et al., 2018). Barsalou et al. (2018) proposed to move beyond the concrete/abstract distinction altogether and to view all concepts within a *situated conceptualization framework*, where representations are multimodal and different kinds of concepts draw on different situations and contexts.

Barsalou et al. (2018) argued that abstract concepts are not well served if we define them based on what they are *not* (i.e., not attached to a physical entity that is perceptible in the real world). According to Barsalou et al.’s (2008) LASS view, multiple systems underlie our knowledge of concepts. LASS focuses on the linguistic and the simulation systems, which interact continuously. In this view, all concepts involve an interaction between the linguistic and perceptual systems (sensory, motor, and emotive). Barsalou et al. argued that words serve as “pointers” to the object, entity, or situation to which they refer. In this way, many of the traditional lexical measures – naming, lexical decision, semantic decision tasks – may not be the best measures of word meaning retrieval, since they involve simple responses to words presented without context and thus may not tap the rich meanings to which those words point. This may be particularly true for abstract concepts which can involve a simulation of an entire situation. A relatively “abstract” concept such as *justice*, when simulated, could parse into a rather concrete situation: a courtroom with a judge, an obvious criminal and victim. Contrarily, when considering the broader context, even an undeniably “concrete” concept such as *table* could evoke a complexity of abstractness when you consider a situation such as “all the stakeholders brought their issues to the *table*”. In this situation there is no object present, but rather a simulation of an abstract situation.

There is evidence that our representations for concepts change with development and experience. A recent analysis showed that children’s emotion concepts are initially quite concrete and then become more abstract across development (Nook et al., 2020). Nook et al. (2020) argued that since emotion concepts do not fit clearly into either abstract or concrete categories, they provide a unique testing ground for understanding development. Nook et al. found, for instance, that younger children tended to provide more situational examples for emotion concepts whereas older children provided increasingly complex definitions including synonyms. Nook et al. also found that acquisition of emotion words extends over a long developmental window: children’s age of comprehension varied considerably across emotions (i.e., understanding at age 4 for *love* but not until age 10 for *calm*). When tapping characteristics beyond simple comprehension, such as definitions, synonyms, and situational examples, Nook et al. found that the variability was even greater, with successful comprehension at age 13 for concepts such as *hate*, *disappointed*, and *love*, but not until age 20 for *proud* and *annoyed*. They found that emotion comprehension plateaued earlier (around age 11) than did more complex emotion abstraction such as defining words and giving synonyms and situational examples, which did not plateau until around age 18.

We think that word association may be a useful next step to investigate children’s vocabulary acquisition in a more contextual way. That is, human knowledge is highly associative, and by examining associations of different word meanings we might gain insight into the way that knowledge is represented. Based on an embodied theory of language development, word meanings are grounded and therefore learned through experience with the world. As such, this theory predicts that word associations would in general be related to the sensory, motor, or emotional experiences associated with the target word. Specifically, Kousta et al. (2011) proposed that emotion grounds the meaning of abstract words. Therefore, in a word association task, abstract words, particularly valenced abstract words, would be more likely to elicit valenced responses whereas concrete target words would likely elicit less valenced responses. This finding would support an embodied hypothesis, specifically that of grounding through emotion for abstract words. Contrarily, the proposal that abstract concepts are grounded in language would predict that the free associations to concrete and abstract cue words will not vary by valence. An examination of the associative structure of word meanings across development could help test these proposals.

## Conclusion

The results of the present study show that even when frequency differences between concrete and abstract words are controlled, abstract words are later acquired. As Gleitman et al. (2005) noted, abstract words are “hard words”. Our results show, however, that this challenge is eased when abstract words are associated with emotion, as measured by valence and interoceptive strength of the word’s referent, or with mouth actions. We take this as evidence that even so-called ‘abstract’ word meanings can benefit from sensorimotor grounding. When that grounding is not present, other mechanisms are important. The task for future research is to further explicate those mechanisms, in order to develop robust theories of children’s vocabulary acquisition, and of semantic representation more broadly.

## Data Availability Statement

Publicly available datasets were analyzed in this study. This data and the analysis scripts can be found here: https://osf.io/cywtv/. MacArthur-Bates Communicative Development Inventory data is available at: http://wordbank.stanford.edu/.

## Author Contributions

LR: conceptualization, writing – original draft preparation, and writing – review and editing. EM: analysis, writing – original draft preparation, and writing – review and editing. PP: conceptualization, and writing – review and editing. All authors contributed to the article and approved the submitted version.

## Conflict of Interest

The authors declare that the research was conducted in the absence of any commercial or financial relationships that could be construed as a potential conflict of interest.

## Footnotes

1 http://wordbank.stanford.edu
